# Potential Applications of Human Viral Metagenomics and Reference Materials: Considerations for Current and Future Viruses

**DOI:** 10.1128/AEM.01794-20

**Published:** 2020-10-28

**Authors:** Tasha M. Santiago-Rodriguez, Emily B. Hollister

**Affiliations:** a Diversigen, Inc., Houston, Texas, USA; University of Bayreuth

**Keywords:** microbiome, mock communities, reference materials, viral metagenomics, virome

## Abstract

Viruses are ubiquitous particles comprising genetic material that can infect bacteria, archaea, and fungi, as well as human and other animal cells. Given that determining virus composition and function in association with states of human health and disease is of increasing interest, we anticipate that the field of viral metagenomics will continue to expand and be applied in a variety of areas ranging from surveillance to discovery and will rely heavily upon the continued development of reference materials and databases. Information regarding viral composition and function readily translates into biological and clinical applications, including the rapid sequence identification of pathogenic viruses in various sample types. However, viral metagenomic approaches often lack appropriate standards and reference materials to enable cross-study comparisons and assess potential biases which can be introduced at the various stages of collection, storage, processing, and sequence analysis. In addition, implementation of appropriate viral reference materials can aid in the benchmarking of current and development of novel assays for virus identification, discovery, and surveillance. As the field of viral metagenomics expands and standardizes, results will continue to translate into diverse applications.

## Abstract (precis)

Viruses are ubiquitous particles comprising genetic material that can infect bacteria, archaea, and fungi, as well as human and other animal cells. Given that determining virus composition and function in association with states of human health and disease is of increasing interest, we anticipate that the field of viral metagenomics will continue to expand and be applied in a variety of areas ranging from surveillance to discovery and will rely heavily upon the continued development of reference materials and databases.

## INTRODUCTION

## 

**FIG 1:**
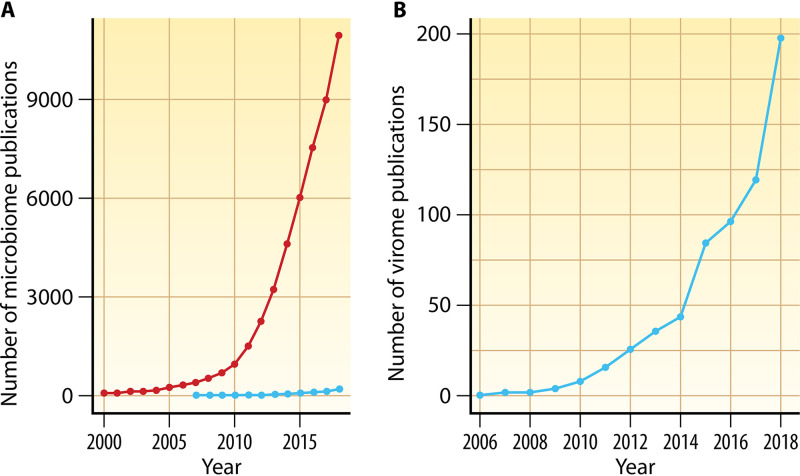
Number of microbiome and virome publications. (A) The number of microbiome publications since 2000 (red line) and virome publications since 2006 (blue line). (B) Expanded graph of the number of viral metagenomic studies since 2006.

Studies targeting the bacterial fraction of the human microbiome have increased in both number and scope during the last decade. By way of example, a recent PubMed search performed using the keyword “microbiome” demonstrates the exponential growth of microbiome studies since 2000 (Fig. 1A). However, the number of studies assessing the viral fraction, or the virome, of various sample types has lagged substantially behind (Fig. 1B). Reasons for the dramatically lower number of virome studies include (i) the intrinsic challenges of studying viruses, including their smaller structural and genome sizes relative to bacteria and other microorganisms, (ii) the high diversity in genome structure and composition (i.e., double-stranded DNA [dsDNA], single-stranded DNA [ssDNA], double-stranded RNA [dsRNA], and single-stranded RNA [ssRNA]), (iii) the lack of a universal gene amenable to amplicon sequencing, as in the case of the 16S rRNA gene for bacteria and archaea, and (iv) biases in sequence databases which emphasize viral pathogens or very well-known bacteriophages (i.e., viruses that infect bacteria), many of which have previously been cultured.

Despite these challenges, viruses are important members of the human microbiome. Viruses are present across the body, at sites, including the gut (1), the skin (2), and the oral cavity (3), and they can inhabit body sites and samples types previously thought to be sterile, including the bladder (4), blood (5), and cerebrospinal fluid (6). Certain viruses can be acquired through birth and continue to be seeded by the maternal bond (7) and shaped by dietary habits (8), as well as intimate contact (9). Viral communities are associated with disease phenotypes, including periodontal disease (10) and inflammatory bowel disease (IBD) (11), and can respond to antibiotic treatment as an indirect response associated with changes in bacterial community composition (12). Viruses, specifically bacteriophages, are now being revisited as a tool to treat antimicrobial-resistant infections and various disease phenotypes associated with toxin-producing bacteria. This was demonstrated in a recent study where bacteriophages infecting cytolysin-producing Enterococcus faecalis strains ameliorated liver disease severity in patients with alcoholic hepatitis (13). Importantly, a number of phage therapy clinical trials have been conducted. For instance, the PhagoBurn study was established to evaluate the treatment of Escherichia coli and Pseudomonas aeruginosa burn wound infections using bacteriophages. It was also the first prospective multicentric, randomized, single blind and controlled phage therapy clinical trial according to both good manufacturing practices (GMP) and good clinical practices (GCP) (https://globalclinicaltrialdata.com/trial/GCT022014-000714-65). Additional clinical trials which seek to identify phage cocktails to treat burn wound infections are under way (https://globalclinicaltrialdata.com/trial/GCT0104323475). Other clinical trials have evaluated bacteriophages as prebiotics, which are defined as indigestible dietary components that promote specific beneficial bacterial species (https://globalclinicaltrialdata.com/trial/GCT0103269617). In addition, viruses can also predict disease risk (14). A recent study characterizing the stool viromes of children at increased risk for type 1 diabetes showed that enterovirus B (EV-B) was one of the most prevalent viruses in the children’s stool samples. The study also found that children with prolonged shedding of the same EV-B serotype had higher odds of developing islet autoimmunity than children negative for EV-B (14). These findings demonstrate the potential applications of viruses for both disease treatment and prediction.

A number of the discoveries highlighted above have been facilitated through the field of viral metagenomics. Studies demonstrating the current and future potential for viral metagenomics revolve around the following key focus areas: (i) understanding the natural history of viruses, particularly virus presence/absence and potential integration/lysis mechanisms, (ii) identifying viral associations with disease, including unexplained illnesses, (iii) identifying potential novel viral relationships with health and disease, (iv) identifying viral associations as risk factors for disease, and (v) using viral metagenomics as a surveillance tool for animal, community, and global health.

## BIASES IN VIRAL METAGENOMICS

**FIG 2:**
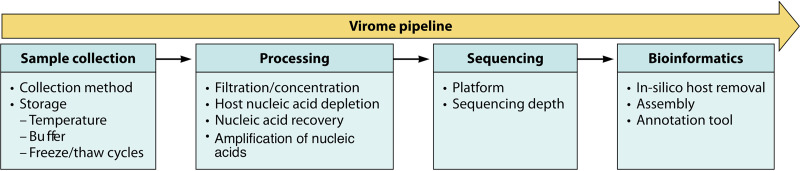
Description of a viral metagenomics pipeline. A viral metagenomics pipeline may include sample collection, processing, sequencing, and bioinformatics. The figure shows several of the steps that may add biases to the results.

Although there are a number of applications for viral metagenomics, biases can be introduced at many points along the process. From sample collection and processing to data generation and analysis, many choices and processes can impact data quality, interpretation, and comparison. A viral metagenomics pipeline usually includes sample collection, sample processing, sequencing, and bioinformatic analyses, similar to a microbiome pipeline (Fig. 2). In the following sections, several of the potential biases introduced at the various steps are discussed.

### Sample collection and storage.

Sample collection practices are often dependent on sample type. For instance, collection methods for samples such as saliva and urine might differ from those for stool due to the nature of the sample matrix and the amount of material needed for downstream processing. Once a sample has been appropriately collected, a major factor that can challenge the recovery of viral nucleic acid and viral community information is storage temperature. Although this has not been widely assessed in the context of viral metagenomics, microbiome studies have shown a significant impact of storage temperature on bacterial diversity and membership (15). Studies assessing individual virus titers can also provide a sense of the impact of storage temperature on virus viability for downstream applications. For instance, a study assessing the viability of bacteriophage MS2 in wastewater found that only 20% of the initial titer was inactivated at 4°C, compared to a reduction of 57% when storing the sample at −80°C after approximately 8 days (16). Tailed phages, such as those infecting Staphylococcus aureus, can be stored at −80°C for long-term preservation as long as a stabilizer is added (17). This usually ensures viability by protecting phage tails. Other viruses, such as hepatitis C, can be inactivated after 5 days at room temperature and after 6 months at 4°C, and can show a decrease of 15.6% after 5 days at −20°C (18). While each virus demonstrates differing inactivation rates depending on the storage conditions used, storage at −80°C is typically considered to be the gold standard for long-term preservation of samples intended for microbiome analyses, and warmer storage temperatures can significantly influence results (15).

Certain studies, particularly those where samples are collected in remote areas or are associated with outbreaks and pandemics, may require preservation buffers if there is no immediate access to a −80°C freezer. For example, RNAlater (Ambion, Inc., Austin, TX, USA), has been shown to preserve tissue samples in a similar manner as stored snap-frozen samples (19). More specific to viruses, OMNIgene.GUT (DNA Genotek, Ottawa, Canada) and its preservation buffer are known to preserve dsDNA viruses, including CrAssphage and bacteriophage T5, in a manner similar to storage at −80°C (20). Experiments have shown a significant decrease in bacteriophage T5, but not in CrAssphage, at room temperature after 14 days without preservation buffers or other stabilization agents (20). Similar experiments have shown the ability of OMNIgene.GUT to preserve both bacteriophage T5 and CrAssphage under different conditions (20). These results indicate that, although some viruses may be persistent across different sample storage conditions, as in the case of CrAssphage, other viruses, such as bacteriophage T5, may be more sensitive to storage temperature, duration, and other storage conditions. It is possible that these findings may also translate to other viruses, including RNA viruses (e.g., severe acute respiratory syndrome coronavirus 2 [SARS-CoV-2], hepatitis C, and influenza viruses), indicating that it is important to select the most suitable stabilization conditions depending on one’s study and its goals. References materials, for instance, may provide insights into the expected outcomes. In addition, future studies are needed to further assess the effect of storage conditions on viruses associated with urine, blood, and cerebrospinal fluid. Importantly, addition of a preservation buffer to maintain virome profiles may not always be necessary when storing samples at −80°C. This may be the case for stool samples, where the majority of the viruses are temperate bacteriophages and have been shown to be resistant to profile changes after long-term preservation at −80°C (21). This may also be explained by the stability in bacterial community composition after long-term storage at −80°C (22).

Increasing numbers of freeze-thaw cycles are known to affect the detection and distribution of members within microbial communities, particularly bacteria and viruses (23). Freeze-thaw cycles are common when samples of interest are utilized multiple times without prior aliquoting and homogenization. Freeze-thaw can also happen during freezer failures or insufficient maintenance of the cold chain during the shipping of the samples to a laboratory facility. This may result in, but is not limited to, changes in viral titer and increased cellular debris, which may, in turn, affect downstream purification steps, including host cell nucleic acid removal (24). Although certain DNA and RNA viruses, including hepatitis B and C viruses, HIV, and SARS-CoV, have been shown to be stable after multiple freeze-thaw cycles involving freezing at −70°C or −80°C followed by thawing in a water bath at 25°C for 1 h (hepatitis B and C) or room temperature (HIV) (25–27), the sensitivity of bacteria to freeze-thaw cycles may have the potential to impact the recovery, diversity, and composition of bacteriophages, particularly prophages (21), and additional work is needed in this space. Certain viruses are directly affected by freeze-thaw cycles, while others are relatively stable. For example, intact influenza virus is dramatically impacted by a single freeze-thaw cycle, reducing the concentration of filaments by almost half (28). In contrast, extracted RNA from influenza H1N1 demonstrates stability for up to 56 days at −80°C or −20°C or up to 9 freeze-thaw cycles (29). Similar experiments should be performed on virus(es) of interest to assess viability and nucleic acid stability preceding molecular analyses. Likewise, the effects of storage conditions and freeze-thaw cycles need to be assessed in novel and emerging viruses.

### Sample processing.

Sample preprocessing for viral metagenomics is crucial. Viral metagenomics protocols may include a filtration/concentration step, where samples are centrifuged to remove any debris. Centrifugation may be followed by serial filtrations using 0.45- and/or 0.22-μm membranes, which aim to remove cells larger than the pore size selected. In addition, filtration may be followed by a concentration step using protein columns, as well as a purification step using cesium chloride (CsCl) gradient ultracentrifugation, for example (9). Other methods include dithiothreitol (DTT) treatment prior to filtration and nucleic acid extraction, as well as CsCl gradient ultracentrifugation after filtration and DNase treatment. Results have shown that while the CsCl gradient ultracentrifugation method outperforms the DTT methods in removing host DNA, DTT methods discriminate less against specific phage species and can yield more DNA (30). Application of one or several of these practices can significantly influence results. For instance, a previous study testing one or several treatments for virus isolation and purification in saliva samples showed that recovery of the vaccinia virus is reduced after centrifugation, as well as when applying filtration through 0.45-μm membranes (31). One possible reason for the loss of the vaccinia virus Western Reserve (WR) strain when using filtration is the large size of the virus, which has dimensions of approximately 360 by 270 by 250 nm. Using 0.22-μm and 0.45-μm membranes may have resulted in virus retention due to the large size of the virus. Studies have suggested pretreatments of the filters that include passing an appropriate buffer, 10% fetal calf serum, veal infusion broth, or bovine albumin prior to filtering to decrease virus retention when using an appropriate-sized filter (32). In the case of bacteriophages, it is essential to remove any bacterial cells using centrifugation and filtration and to apply DNase and RNase treatments before nucleic acid extraction to remove extracellular nucleic acids that may be host associated. This is particularly important because phage genes share a degree of homology with bacterial genes and, thus, may affect downstream data interpretation.

The application of one or several of these steps may be useful, particularly when working with samples that originate from sites with a high level of host contamination. Saliva, blood, biopsy specimens, skin swabs, and cerebrospinal fluid are several sample types known to have a high degree of host burden. In such cases, host cell removal may aid in obtaining accurate profiles and increase the viral signal. Alternatively, enrichment methods for specific viruses have shown promising results. For instance, ViroCap was designed to enrich nucleic acids from 34 viral families infecting vertebrate hosts (33). Other examples include VirCapSeq-VERT, targeting over 200 viral taxa (34), hybrid-capture target enrichment using PCR-generated capture probes (35), and more recently, multiplex amplicon- and hybrid capture-based sequencing with ultrahigh-throughput metatranscriptomics for SARS-CoV-2 studies (36). However, in cases of low-volume samples, as in the case of skin swabs, applying several of these steps may result in sample loss. In such cases, deeper sequencing and postsequencing host sequence removal may provide actionable results with the caveat of lower evenness (e.g., Shannon diversity index), as in the case of skin swab samples (2). In other cases, deeper sequencing may not be required due to the low diversity of the samples but may be useful to increase genome coverage (37). Another study, evaluating 16 different concentration, extraction, and purification protocols, showed that tangential flow filtration (TFF), pyrophosphate in combination with sonication, and ultracentrifugation in a sucrose gradient yielded significantly greater numbers of virus-like particles (38). While there is no method or combination of methods known to provide optimal results, sample preprocessing would need to be evaluated as needed for the particular virus(es) of interest, although this may add additional biases.

### Nucleic acid extraction and amplification of viral nucleic acids.

Historically, viral nucleic acid extraction methods were developed according to the sample type and the virus(es) being targeted (39, 40). Commercially available methods now facilitate viral nucleic acid extraction, and a number of them are intended for specific sample types. For instance, a study evaluating the nucleic acid extraction efficiency from HeLa cells spiked with four viruses, including the double-stranded DNA Epstein-Barr virus, double-stranded RNA reovirus 3, single-stranded RNA feline leukemia virus, and respiratory syncytial virus, using 11 commercially available extraction kits based on silica membrane column, magnetic bead, and precipitation-based extractions found that dual extraction methods may provide improved sensitivity for recovering nucleic acids from viruses with specific biochemical and biophysical characteristics (41). Another study, testing three nucleic acid extraction methods in biofilms, including chloroform, tetrasodium pyrophosphate in combination with sonication, and DTT, showed various DNA yields that may be explained by the mode of action of the chemicals used. For instance, chloroform may denature the lipid envelopes surrounding viral capsids, as in the case of viruses from the *Phycodnaviridae* family, including, for instance, MpoVs, which infect Micromonas polaris (38, 42). Results indicate that nucleic acid extraction methods would need to be tested independently, ideally using reference materials, which may help in understanding the biases that could be introduced at this stage.

Viral nucleic acid extraction may result in low to moderate yields, depending on the method(s) used. For this reason, whole amplification of the nucleic acids may be considered. One method, known as multiple displacement amplification (MDA), can result in significantly higher nucleic acid yields, as it utilizes a high-fidelity enzyme (43). Other amplification methods include sequence-independent single-primer amplification (SISPA), which is a primer-initiated technique that requires target sequence modification preceding the logarithmic amplification of the DNA (44). However, random amplification methods may result in the overrepresentation of certain viruses, including ssDNA viruses. For instance, a study assessing the effect of MDA found the overrepresentation of bacteriophage M13 in a mock community composed of the seven DNA viruses bacteriophage lambda, vaccinia virus, phage phi29, adenovirus, bacteriophage M13, mouse minute virus p, and a porcine circovirus (31). This suggests that, while amplification methods aid in obtaining higher nucleic acid yields, results should be carefully interpreted, particularly in terms of abundances.

### Sequencing technology.

Although Illumina sequencing has dominated the field of metagenomics, other sequencing platforms are also currently being used (e.g., the Ion semiconductor sequencing platform). However, combining the outputs of various sequencing platforms may not be ideal when performing certain meta-analyses (i.e., analyses combining data sets from different studies). In the case of viromes, however, utilizing Illumina versus Ion Torrent sequencing may not affect the diversity output. This has been noted for cerebrospinal fluid, where viral alpha and beta diversity were not significantly altered by the sequencing platform (6). Additional studies are needed to understand the effect of the sequencing platform on more complex samples (e.g., stool and soil samples). High-throughput sequencing is also increasingly being applied to clinical specimens aiming to identify specific pathogenic viruses. A study assessing the effect of the sequencing platform and the sequencing kit using clinical specimens positive for various enteroviruses and polioviruses showed that the number of viral reads depends on the type of virus, as well as the sequencing platform and sequencing kit. For instance, the number of enterovirus reads did not seem to be affected by the sequencing platform or sequencing kit, but the number of poliovirus reads was affected (45). The virus coverage was also affected by the sequencing platform and sequencing kit, where the genome coverage was highest when using the Illumina MiSeq 500 v2 kit compared to the Ion Torrent PGM kits (45). This seemed to be dependent on the higher number of reads generated by the Illumina MiSeq 500 v2 kit compared to the Ion Torrent PGM kits. Ideally, further studies assessing the effect of the sequencing platform and sequencing kit may include viral reference materials.

### Bioinformatic analyses: host removal and assembly.

Bioinformatic analyses in viral metagenomics may include several steps preceding annotation. As with many microbiome studies, viral metagenomics research is accompanied by the *in silico* removal of host sequence (i.e., human, animal, or plant) postsequencing. This helps to ensure that the analysis consists primarily of viral sequences, which in turn, may significantly reduce the amount of computer power and time to process results. Following host sequence removal, viral metagenomic sequence analysis approaches may include viral sequence assembly, and depending on the annotation tool used, differing results can be acquired as a function of the assembler used and the degree of success achieved in the assembly process. This makes metagenomic assembly particularly challenging for virome data, as it may result in fragmented assemblies and, consequently, poor annotations. A previous study tested the ability of 16 different tools for sequence assembly in several sample types, including a viral mock community comprising 12 viral genomes, 10 of which were at equal abundance (9.82% relative abundance/virus) and 2 of which were ssDNA genomes (0.92% relative abundance/virus) (46) (Table 1). For this mock community, specifically, particular assemblers, including CLC, Geneious, SPAdes, and VICUNA, were able to detect all 12 genomes. However, a number of false positives (i.e., alignment to a number of reference genomes) were also identified. Other assemblers, particularly, Velvet and MetaVelvet, generated no false positives but failed to assemble three genomes. In contrast, ABySS generated a large number of false positives and failed to assemble four to six genomes, depending on the k-mer setting used. The assemblers IDBA UD and Ray Meta outperformed the other assemblers with an equal number of contigs to genomes, followed by MEGAHIT and SPAdes (46). This illustrates the biases introduced when assembling viral sequences and the ability of reference materials to determine the performance of assembly tools. Reference materials may be used in parallel to the sample of interest to determine the effect of assemblers in detecting the expected viruses, as well as the specificity, including the number of false positives and the number of false negatives. This may also need to be applied as novel assemblers become available.

**TABLE 1:** Assembler performance^*a*^

Assembler	No. of false positives	No. of false negatives	No. of true positives	No. of contigs	Sensitivity (%)	Source
ABySS (v2.0.2) (*k*-mer 63)	52	4	8	61	66.67	http://www.bcgsc.ca/downloads/abyss/
ABySS (v2.0.2) (*k*-mer 127)	50	6	6	56	50	http://www.bcgsc.ca/downloads/abyss/
CLC (v5.0.5)	1,143	0	12	1,299	100	https://www.qiagenbioinformatics.com/products/clc-assembly-cell/
Geneious (v11.0.3)	53	0	12	65	100	https://www.geneious.com/features/assembly-mapping/
IDBA UD (v1.1.1)	0	0	12	12	100	https://i.cs.hku.hk/~alse/hkubrg/projects/idba_ud
MEGAHIT (v1.1.1-2)	0	0	12	13	100	https://github.com/voutcn/megahit
MetaVelvet (v1.2.02)	0	3	9	26	75	https://metavelvet.dna.bio.keio.ac.jp/
MIRA (v4.0.2)	0	0	12	89	100	https://sourceforge.net/projects/mira-assembler/files/
Ray Meta (v2.3.0)	0	0	12	12	100	http://denovoassembler.sourceforge.net/
SOAPdenovo2 (v2.04)	2	0	12	23	100	https://sourceforge.net/projects/soapdenovo2/
SPAdes (v3.10.0)	0	0	12	14	100	http://cab.spbu.ru/software/spades/
SPAdes meta (v3.10.0)	0	0	12	14	100	http://cab.spbu.ru/software/spades/ (variation of SPAdes applied with flag)
SPAdes sc	1,513	0	12	1,527	100	http://cab.spbu.ru/software/spades/ (variation of SPAdes applied with flag)
SPAdes sc careful	0	0	12	15	100	http://cab.spbu.ru/software/spades/ (variation of SPAdes applied with flag)
Velvet (v1.2.10)	0	3	9	26	75	https://www.ebi.ac.uk/~zerbino/velvet/
VICUNA (v1.3)	4,969	0	12	5,385	100	https://github.com/broadinstitute/mvicuna

a Performance was previously evaluated using, among many factors, the number of false positives, false negatives, and true positives, the number of contigs, and sensitivity. Links to the assembler sources are also shown. Modified from reference 46.

### Bioinformatic analyses: annotation tools.

Annotation tools can also impact the outcomes of a viral metagenomics study. A number of annotation tools specific for viruses are available, which can be alignment- or k-mer-based (47). Since a number of annotation tools will continue to be available, it is important to evaluate the most suitable annotation tool based on specific research needs. For instance, a study assessing the viral content of benthic deep-sea samples using BLAST, MG-RAST, NBC, VMGAP, MetaVir, and VIROME showed that the BLAST tools, followed by MetaVir and VMGAP, provided the most reliable results. In addition, while tBLASTx, MetaVir, VMGAP, and VIROME showed a similar efficiency of sequence annotation, MetaVir and tBLASTx identified a higher number of viral strains (48). Another tool, known as VirMAP, employs multiple methods that include a combination of *de novo* assembly and mapping-based strategies to taxonomically classify sequences (49). While these tools possess a number of advantages, most are database reliant and not necessarily suitable for novel virus discovery. For this reason, another more recent tool known as VIBRANT (Virus Identification By iteRative ANnoTation) enables both virus identification and discovery utilizing machine learning and protein similarity approaches. Importantly, VIBRANT highlights viral auxiliary metabolic genes and metabolic pathways, which are usually not covered by other annotation tools (50). Another tool, known as DeepVirFinder, is a reference- and alignment-independent machine learning method for identifying viral sequences in metagenomics using deep learning (51). Performance of current and future viral taxonomic and functional annotation tools can be assessed using reference materials.

## ASSESSING BIASES IN VIRAL METAGENOMICS

Although several approaches exist for assessing the biases introduced at one or more of the steps described above, reference materials represent a standardized manner to benchmark pipelines, reagents, and bioinformatic analyses. Reference materials also represent an approach to assess biases that could be introduced even after standardization (i.e., technician variation, shipping issues, power shortages, etc.). However, the practice of including reference materials arose relatively recently as an approach to address the reliability of Illumina sequencing as a substitute for 454 sequencing (52). Specifically, the term “mock community” arose to refer to a mix of bacterial DNA (52) and is currently used to refer to a mix of bacterial or fungal cells or DNA, as well as viral particles or nucleic acids in specific concentrations. A number of these reference materials include mock communities composed of organisms with diverse structural (e.g., cell wall and morphology) and genomic characteristics (e.g., GC content and genome size), mock communities mimicking various body sites, organisms intended to be spiked into a sample, and homogenized stool samples.

Although the importance of reference materials is increasingly being acknowledged, there is room for improvement in the field. For instance, only 30% of articles published in 2018 in two broadly read and well-cited microbiome and microbiology journals reported a negative control, and only 10% reported results from positive controls (53). This suggests that there is the need to implement and report the results of both negative and positive controls in microbiome studies. Although such controls may include a variety of materials, reference materials, specifically, can be used as positive and negative controls; however, reference materials, including mock communities, are not implemented on a regular basis in microbiome studies, even though they are available through research and academic institutions, as well as commercial facilities. Most reference materials available are intended for microbiome analysis targeting the bacterial fraction, and these have been shown to be helpful in benchmarking nucleic acid extraction methods (54), sequencing platforms and sequencing kits (55), assemblers, and taxonomic and functional classification tools (56, 57). A limited number of reference materials have been developed for viral metagenomics research, and it is anticipated that additional materials may be developed as viral metagenomics applications experience broader adoption.

Viral reference materials, like bacterial and fungal reference materials, aid in assessing biases introduced into a virome pipeline, as described above. Commercially available reference materials, particularly, viral mock communities from the American Type Culture Collection (ATCC, Manassas, VA, USA), can be used to identify and potentially quantify biases introduced at particular stages of a viral metagenomics pipeline, as highlighted in the previous sections. ATCC’s viral mock communities are composed of equal concentrations of either nucleic acids from five different viruses or whole viruses. The mock communities possess dsDNA viruses, including enveloped viruses (herpesvirus) and unenveloped viruses (adenovirus). The mock communities also possess RNA viruses, including a positive-sense ssRNA virus (Zika virus), negative sense ssRNA virus (influenza B virus and human respiratory syncytial virus), and a dsRNA virus (reovirus 3) (58).

## POTENTIAL FUTURE APPLICATIONS OF VIRAL METAGENOMICS AND AREAS FOR GROWTH

**FIG 3:**
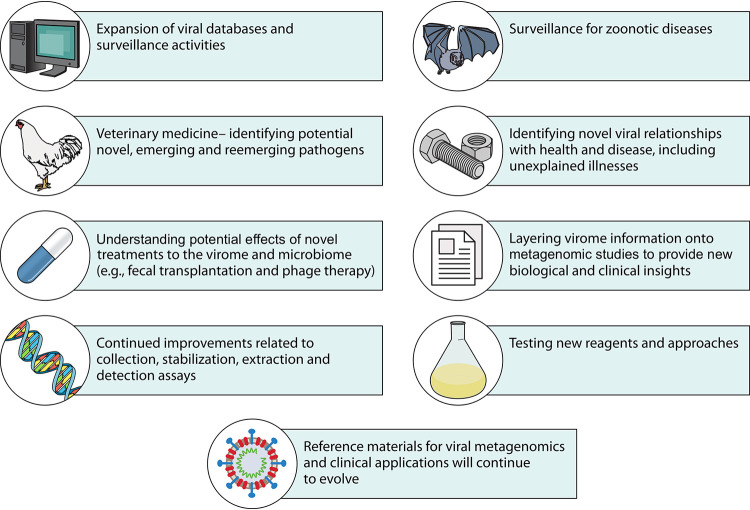
Potential applications of viral metagenomics in ongoing and future fields related to database expansion, surveillance, identification of viral relationships with health and disease, and reagent development, among others.

Although viral metagenomics has been widely used for virome characterization in various sample types, the approach can be leveraged in a number of different ways to address current and future virus discovery and tracking. These ways include but are not limited to (i) expansion of viral databases through virus discovery efforts, (ii) surveillance activities, particularly among wildlife reservoirs or in the context of SARS-COV-2 in wastewater, (iii) identification of emerging and reemerging pathogens in veterinary medicine, (iv) identification of novel viral relationships in health and disease, including unexplained illnesses, (v) understanding the potential effects of novel treatments (e.g., fecal transplantation and phage therapy) on the virome and microbiome, (vi) layering virome information onto metagenomic studies to provide new biological and clinical insights, (vii) continued improvements related to sample collection, stabilization, extraction, and detection assays, (viii) testing new reagents and approaches, and (ix) development of reference materials for viral metagenomics and clinical applications (Fig. 3).

The numerous potential (future and ongoing) applications of viral metagenomics are increasingly being realized. In the areas of discovery, for instance, viral metagenomics has shown increasing value. A study evaluating the use of viral metagenomics for the identification of viruses in bats showed that while only <1.0% of the reads are viral, 83% are bacteriophage, insect-borne viruses, and plant viruses, and 14% represent mammalian viruses (59). In addition, while genome segments within a number of the mammalian viruses shared a high degree of homology to known viruses, a number of these segments differed, suggesting that bats possess a number of viruses that remain to be characterized (59). This approach may also translate to other animals, particularly those harboring potential zoonotic viruses. More recent evidence has also demonstrated the value of viral metagenomics in tracing potential recombination hosts for SARS-CoV-2. Although it has been demonstrated that bats may have been the original reservoirs of the novel coronavirus, genomic evidence has also shown that the pangolin may have served as an intermediate host (60). This was also demonstrated using viral metagenomics, where a coronavirus strain similar to the human strain was identified from metagenomic data sets (61).

In addition to the application of viral metagenomics for the discovery and identification of viruses, including SARS-CoV-2, the potential for surveillance activities remains largely unexplored. Viral surveillance activities in various sample types rely largely on PCR- or panel-based techniques, which exhibit a number of advantages, including specificity and speed. However, one drawback is that surveillance activities based on PCR- or panel-based techniques may be limited to the virus(es) of interest. Viral metagenomics, on the other hand, exhibits the additional advantage of the identification of a number of different viruses simultaneously, including novel viruses. This suggests that viral metagenomics may be added to the toolbox of current methods for virus surveillance in clinical and environmental samples (62). Similarly, viral metagenomics has an increasing potential in virus surveillance in novel treatment methods. For instance, fecal transplantation has been shown to be effective in treating Clostridium difficile infections, but the transmission of viruses from donor to recipient remains largely unexplored. A study assessing the viral contents after fecal transplantation from a donor to three pediatric recipients found that most of the viruses that were transmitted were bacteriophages (63), which is in agreement with a previous study assessing the viral contents of chemostat systems (64). This approach may also apply to the identification and surveillance of other potentially pathogenic viruses in donor and recipient stool samples. Surveillance activities using sequencing approaches may also be applied in undercharacterized environments, such as the built environment (e.g., hospitals and schools). Most studies characterizing the built environment focus on the bacterial fraction (65). Thus, viral metagenomics studies of the built environment in developed and developing countries, and in both rural and urbanized regions, are still needed. Results may provide information regarding the risk of infection of viral pathogens, as well as transmission dynamics and routes of transmission (e.g., airborne, fomite, and water routes) in various built environments (65). In addition, a number of these built environments possess a high prevalence of multidrug-resistant bacteria, which represent a risk to public health. Bacteriophages have, therefore, been suggested as a means to control multidrug-resistant bacteria in built environments (65). In this case, sequencing approaches may aid in identifying any prophages or prophage remnants in the multidrug-resistant bacterial genomes, which often result in superinfection resistance.

Another area where viral metagenomics may have potential future applications and the potential to develop further is in identifying novel and broad viral relationships in health and disease, including potentially unexplained illnesses. For instance, a study applying high-throughput sequencing in low-biomass samples, including cerebrospinal fluid, blood, and throat swabs found that viruses were identified in 32% of the samples (66). In parallel, conventional virus diagnostic tests were performed, and in multiple cases, the identified viruses were not included in the selected routine diagnostic tests (66). Interestingly, application of viral metagenomics resulted in the adjustment of a subject’s treatment after exclusion of a viral infection (66).

Another potential application of viral metagenomics is the continued development and refinement of viral reference materials. Development of novel viral reference materials may aid to determine biases that can be introduced when testing current and novel reagents for viral nucleic acid extraction or conversion of RNA to cDNA, for instance. In addition, development of novel viral reference materials can be used to validate current and novel surveillance assays (e.g., quantitative PCR [qPCR] assays for the detection of SARS-CoV-2 in sewage and environmental samples). Novel viral reference materials can also be used to validate a metagenomic assay, starting from sample collection and proceeding down to data analysis. In many cases, when viral reference materials represent the original virus strain, it could be an opportunity to track virus evolution. Moving forward, we anticipate that a number of viral reference materials will continue to be added to the collection of those currently available.
